# Biophysical Characterization of a Carotenoprotein from Marine Sponge *Tedania ignis* Reveals Pigment-Dependent Stability and Antibiotic Interactions

**DOI:** 10.3390/md24030118

**Published:** 2026-03-21

**Authors:** Philippe Lima Duarte, Paulo Anderson Paiva Martins, Jéssica de Assis Duarte, Manoel Ferreira da Costa Filho, Ellen Araújo Malveira, Celso Shiniti Nagano, Alexandre Holanda Sampaio, Edson Holanda Teixeira, Rômulo Farias Carneiro, Mayron Alves de Vasconcelos

**Affiliations:** 1 Laboratório de Biotecnologia Marinha—BioMar-Lab, Departamento de Engenharia de Pesca, Universidade Federal do Ceará, Campus do Pici s/n, bloco 871, Fortaleza 60440-970, CE, Brazil; philippe_lima19@hotmail.com (P.L.D.); pauloandersonmk@gmail.com (P.A.P.M.); jessicaaduarte2@gmail.com (J.d.A.D.); manoelcostavy@gmail.com (M.F.d.C.F.); naganocs@gmail.com (C.S.N.); alexholandasampaio@gmail.com (A.H.S.); 2 Laboratório Integrado de Biomoléculas—LIBS, Departamento de Patologia e Medicina Legal, Universidade Federal do Ceará, Monsenhor Furtado, s/n, Fortaleza 60430-160, CE, Brazil; ellenmalveira11@gmail.com (E.A.M.); edson@ufc.br (E.H.T.); 3 Faculdade de Educação de Itapipoca, Universidade Estadual do Ceará, Itapipoca 62500-000, CE, Brazil

**Keywords:** carotenoprotein, marine sponge, carotenoid–protein interaction, antibiofilm activity, antibiotic synergy

## Abstract

Carotenoproteins from marine sponges represent an underexplored class of pigment–protein complexes with distinctive structural and functional properties. Here, we report the isolation and biophysical characterization of a blue carotenoprotein from the sponge *Tedania ignis*, termed Ti-CP. The protein was purified and shown to consist of two closely related isoforms with molecular masses of approximately 27–29 kDa. Reverse-phase chromatography enabled separation of the apoprotein (ApoTi-CP) and its associated carotenoids, which were identified as oxygenated carotenoids consistent with astaxanthin and mytiloxanthin. Circular dichroism analysis revealed that both Ti-CP and ApoTi-CP are dominated by β-sheet secondary structure and display highly similar conformational profiles. In contrast, dynamic light scattering demonstrated that carotenoid binding is critical for protein stability, as the native form exhibited a compact and monodisperse organization, whereas ApoTi-CP showed pronounced aggregation. Isothermal titration calorimetry revealed that Ti-CP, but not ApoTi-CP, interacts with tetracycline, oxacillin, and streptomycin, indicating that pigment-mediated stabilization modulates ligand binding. Both Ti-CP and ApoTi-CP reduced bacterial viability and biofilm formation in a strain-dependent manner and enhanced antibiotic activity, including synergistic effects against resistant bacteria. Together, these results provide a comprehensive description of a previously uncharacterized sponge carotenoprotein and highlight the dual role of carotenoids in structural stabilization and antimicrobial modulation, reinforcing the biotechnological relevance of marine pigment–protein complexes.

## 1. Introduction

Carotenoids are ubiquitous isoprenoid pigments that play central roles in pigmentation, photoprotection, and antioxidant defense across all domains of life [1,2]. However, their hydrophobicity limits their solubility, stability, and direct biotechnological use in aqueous systems. In nature, this limitation is overcome through their association with specific binding proteins, forming carotenoproteins that stabilize carotenoids and modulate their physicochemical and biological properties [3]. These protein–pigment complexes enable carotenoids to function in diverse contexts, ranging from light harvesting and photoprotection to coloration and redox regulation [4,5].

Carotenoproteins have been structurally characterized in only a few biological systems, notably crustaceans and cyanobacteria [6,7]. The best-known example is β-crustacyanin from lobsters, which binds astaxanthin (AXT) into a hydrophobic pocket and shifts its absorption spectrum, generating the characteristic blue coloration [8]. Recently, additional carotenoproteins have been identified in cyanobacteria and marine sponges, revealing that unrelated protein scaffolds have independently evolved the ability to bind carotenoids [9,10]. Nevertheless, despite these advances, the molecular diversity, oligomeric organization, and functional roles of carotenoproteins, particularly in marine sponges, remain largely unexplored.

Marine sponges are exceptionally rich sources of bioactive metabolites and unusual proteins, yet carotenoproteins from sponges have been only sporadically reported. To date, only a few sponge carotenoproteins have been isolated and partially characterized. For instance, detailed studies have been reported mainly for species of the genus *Haliclona*, including two characterized carotenoproteins from *Haliclona* sp., one of which has had its three-dimensional structure resolved. In both cases, the protein complexes were associated with the carotenoids AXT and mytiloxanthin (MXT) [9,10]. In *Haliclona caerulea*, a carotenoprotein (H-3) has been described as a lectin capable of inducing hemagglutination while also exhibiting specific binding to AXT. Notably, the H-3 carotenoprotein demonstrated antioxidant activity and anticancer potential, highlighting the functional versatility of these complexes [11,12,13].

At the molecular level, the primary structures of sponge carotenoproteins consistently show homology to ependymins, a family of glycoproteins originally identified in the brains of teleost fish and historically associated with long-term memory processes [14,15]. This evolutionary and structural relationship suggests that sponge carotenoproteins may belong to a broader class of ependymin-related proteins (ERP) adapted to bind hydrophobic ligands such as carotenoids. This information is particularly relevant because carotenoproteins display hydrophobic pockets capable of binding not only pigments but potentially other small hydrophobic molecules [14], suggesting that they could act as natural carrier systems with biotechnological relevance.

Here we report the isolation and characterization of a new carotenoprotein from the marine sponge *Tedania ignis*, termed Ti-CP. We demonstrate that Ti-CP exists as a pigment-bound native form and as a pigment-free apoprotein, with the carotenoid acting as a critical structural stabilizer. Beyond its biophysical characterization, we show that both Ti-CP and its apoform ApoTi-CP interact with antibiotics, inhibit the formation of bacterial biofilms, and enhance antibiotic activity.

## 2. Results

### 2.1. Purification of Ti-CP

The carotenoprotein Ti-CP was efficiently enriched by ammonium sulfate precipitation, with the 60–90% saturation fraction displaying an intense blue coloration, indicating a strong enrichment of the pigment-binding protein. This fraction was subsequently subjected to anion-exchange chromatography, where Ti-CP was retained on the column and eluted with 150 mM NaCl in 50 mM Tris buffer, pH 7.6 (Figure S1A). The eluted fractions exhibited a distinct blue color (Figure S1B), confirming that the pigment remained associated with the protein throughout the purification process.

SDS-PAGE analysis of these fractions revealed a broad protein band centered at approximately 25 kDa (Figure S1C).

### 2.2. Molecular Mass and Purity Measure

The native molecular mass of Ti-CP was estimated by size-exclusion chromatography (SEC), yielding an apparent molecular mass of approximately 27 kDa (Supplementary Figure S2A), suggesting a monomeric state. Elution was monitored by photodiode array (PDA), which revealed a pronounced absorption signal in the visible region with a maximum around 620 nm (Supplementary Figure S2B).

This spectral feature is characteristic of protein-bound carotenoids and confirms that the blue chromophore remains tightly associated with the protein under native conditions. The coincidence of the 280 nm protein peak with the visible absorption further indicates that the pigment and protein co-elute as a single molecular species, consistent with a stable carotenoprotein complex.

In SEC, a single major peak was observed, accounting for approximately 98.77% of the total protein, as determined by chromatographic peak integration. This result indicates a high degree of purity of the protein under study.

### 2.3. Separation of the Carotenoid by Reverse-Phase Chromatography

**Figure 1:**
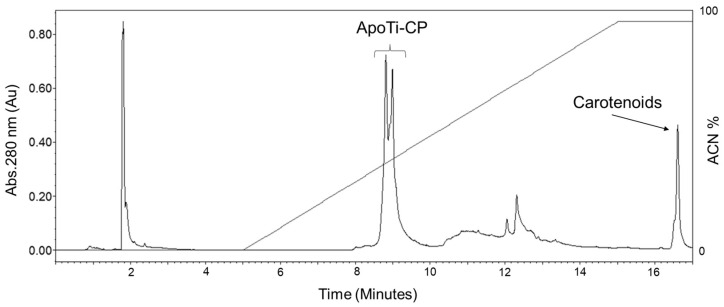
RPC profile of Ti-CP dissociation. RPC profile showing the separation of the apoprotein (ApoTi-CP) and the carotenoid moiety after dissociation of the native carotenoprotein (Ti-CP). Elution was carried out using a linear gradient of organic solvent, and chromatographic profiles were monitored by UV absorbance. The early-eluting peak corresponds to the apoprotein fraction (ApoTi-CP), reflecting its lower hydrophobicity, whereas the late-eluting peak corresponds to the hydrophobic carotenoid fraction released from the native complex.

To dissociate the pigment from Ti-CP, the purified carotenoprotein was subjected to reverse-phase chromatography (RPC) on a C8 column. Under these conditions, the protein and the carotenoid were efficiently resolved (Figure 1). The apoprotein eluted within the acetonitrile (ACN) gradient, whereas the pigment was retained and eluted only at the end of the run, consistent with its strong hydrophobic character. The apoprotein-containing fractions were colorless, while the pigment fraction exhibited an intense orange coloration, indicating the release of the carotenoid from the protein matrix and a pronounced change in its optical properties relative to the native blue complex.

**Figure 2:**
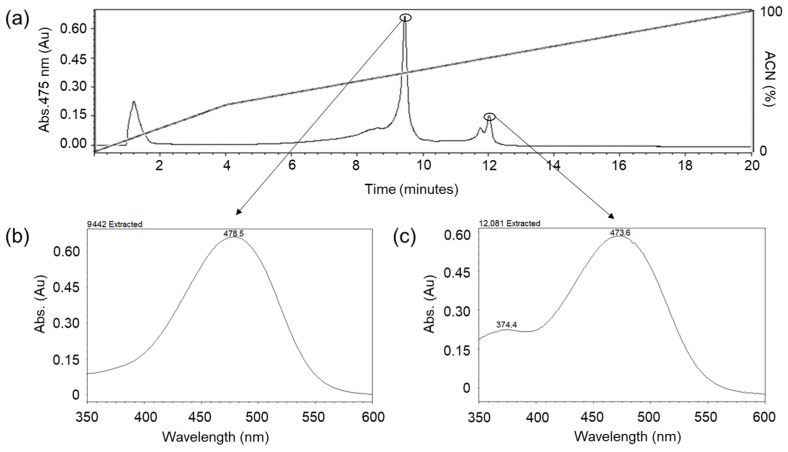
Reverse-phase separation and spectral characterization of carotenoids associated with Ti-CP. The pigment fraction released from Ti-CP was further resolved by reverse-phase chromatography on a C18 column using an organic solvent gradient (upper panel). (**a**) Elution was monitored by PDA detection, revealing two well-resolved pigment peaks. PDA spectra extracted at the corresponding retention times are shown in the lower panels. The dashed line indicates the organic solvent gradient (mobile phase) applied during the reverse-phase chromatographic separation. (**b**) The first pigment peak (RT ≈ 9.44 min) exhibited a maximum absorption at 478.5 nm. (**c**) The second peak (RT ≈ 12.08 min) displayed a main absorption maximum at 473.6 nm together with an additional band at ~374.4 nm, a spectral feature characteristic of cis-configured carotenoids.

The isolated carotenoid fraction was subsequently analyzed by a second RP separation on a C18 column coupled to a PDA detector (Figure 2a). This analysis revealed the presence of two chromatographically resolved species, each displaying a distinct absorption spectrum in the visible range (Figure 2b,c).

The PDA spectra showed two absorption maxima characteristics of oxygenated carotenoids, indicating that the pigment fraction is composed of two chemically distinct carotenoid species rather than a single homogeneous compound.

### 2.4. Secondary Structure Analysis by Circular Dichroism (CD)

**Figure 3:**
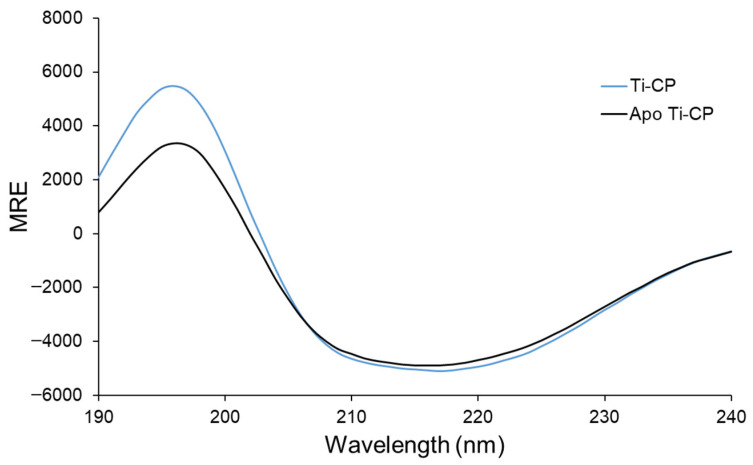
Far-UV circular dichroism spectra of Ti-CP and ApoTi-CP. Far-UV CD spectra (190–240 nm) of the native carotenoprotein Ti-CP (blue line) and its pigment-free form ApoTi-CP (black line). Both spectra display a positive band near 195 nm and a negative minimum between 215 and 220 nm, characteristic of proteins dominated by β-sheet secondary structure. The high similarity between the spectra indicates that removal of the carotenoid does not induce major changes in the overall secondary structure of the protein.

Following pigment removal, both the native Ti-CP and ApoTi-CP were analyzed by far-UV circular dichroism (Figure 3). The CD spectra of Ti-CP displayed a positive band near 195 nm and a negative minimum between 215 and 220 nm, a spectral signature characteristic of proteins dominated by β-sheet secondary structure.

Both protein forms exhibited highly similar CD profiles, indicating that removal of the carotenoid does not induce major alterations in the global secondary structure. Deconvolution of the spectra revealed that native Ti-CP is composed of approximately 9% α-helix, 35% β-sheet, 21% turns, and 35% disordered structures, whereas ApoTi-CP contained 7% α-helix, 36% β-sheet, 22% turns, and 35% disordered regions. These closely matching values demonstrate that the carotenoid does not play a dominant role in maintaining the secondary structure architecture of the protein, despite its strong impact on other biophysical properties.

Analysis of the thermal transition using the Thermal Denaturation tool implemented in the BestSel platform yielded a melting temperature (Tm) of 49.3 °C (Figure S3).

### 2.5. Hydrodynamic Behavior of Ti-CP and ApoTi-CP

**Figure 4:**
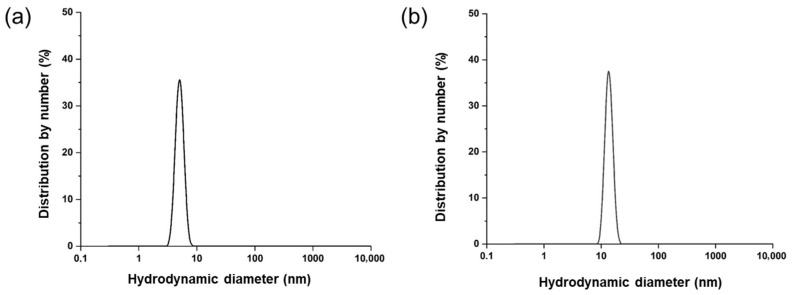
Hydrodynamic size distribution of Ti-CP determined by DLS. Number-weighted hydrodynamic diameter distributions obtained by dynamic light scattering (DLS) for the native carotenoprotein Ti-CP (**a**) and its carotenoid-depleted form, ApoTi-CP (**b**). Measurements were performed at 20 °C using protein samples prepared in ultrapure water. Size distributions were calculated from the autocorrelation functions using the general-purpose analysis model. The profiles illustrate the hydrodynamic behavior of the intact pigment–protein complex compared with its apoprotein form following carotenoid removal.

Dynamic Light Scattering (DLS) measurements revealed marked differences in the hydrodynamic behavior of the Ti-CP and ApoTi-CP (Figure 4). When expressed as number-weighted distributions, Ti-CP displayed a single, narrow population with a hydrodynamic diameter centered at approximately 7–8 nm, indicative of a monodisperse and well-organized system in solution (Figure 4a). No significant populations corresponding to larger aggregates were detected, suggesting high colloidal stability under the experimental conditions.

Considering the molecular mass determined by mass spectrometry (27–29 kDa per subunit), the observed hydrodynamic diameter of Ti-CP is larger than expected for a monomeric globular protein, suggesting the presence of a compact oligomeric assembly in solution.

In contrast, the removal of the carotenoid profoundly affected the hydrodynamic properties of the protein. ApoTi-CP displayed polydisperse populations extending to larger diameters, consistent with self-association and aggregation processes (Figure 4b).

### 2.6. Mass Spectrometric Analysis of Ti-CP and Its Associated Carotenoids

**Figure 5:**
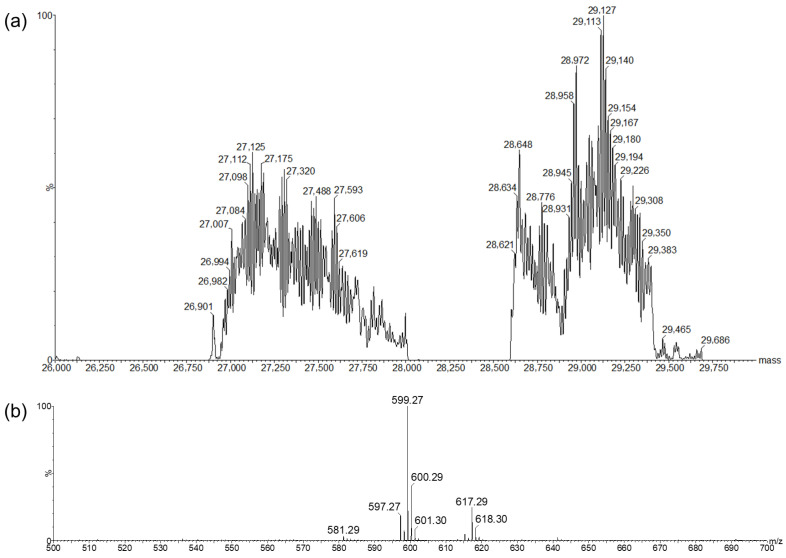
Mass spectrometric analysis of Ti-CP. (**a**) Mass spectrometric analysis of the native carotenoprotein (Ti-CP), revealing two main protein populations with average molecular masses centered at approximately ~27.0 kDa and ~29 kDa, consistent with closely related molecular observed in SDS-PAGE. (**b**) ESI-MS analysis of the pigment fraction released from Ti-CP after dissociation, showing the *m*/*z* signals corresponding to the associated carotenoid species.

The fractions corresponding to ApoTi-CP and the isolated carotenoid were analyzed by Electrospray Ionization Mass Spectrometry (ESI-MS) in positive ion mode to determine their molecular masses (Figure 5).

Analysis of the protein fraction revealed the presence of two distinct molecular species with deconvoluted masses of approximately 27 kDa and 29 kDa, indicating that Ti-CP exists as two closely related isoforms in the native sample. Each of these species generated a series of multiply ions, consistent with intact proteins and suggesting heterogeneity that may arise from amino acid variation and/or post-translational modifications, i.e., glycosylation (Figure 5a).

In parallel, ESI-MS analysis of the pigment fraction revealed a dominant ion at *m*/*z* ~599, accompanied by signals at *m*/*z* ~581 and ~617 (Figure 5b). This pattern is consistent with the presence of an oxygenated carotenoid, MTX (MW 598.86), detected as a protonated species and related ions formed by neutral loss or gain of 18 Da, attributable to dehydration and hydration processes during ionization. In addition, a second ion population centered at *m*/*z* ~597 was detected, consistent with the presence of a second carotenoid species, astaxanthin (AXT, MW = 596.85). Together, these data indicate that Ti-CP associates with at least two distinct carotenoids, consistent with the chromatographic and spectroscopic analyses.

### 2.7. Isothermal Titration Calorimetry (ITC)

**Figure 6:**
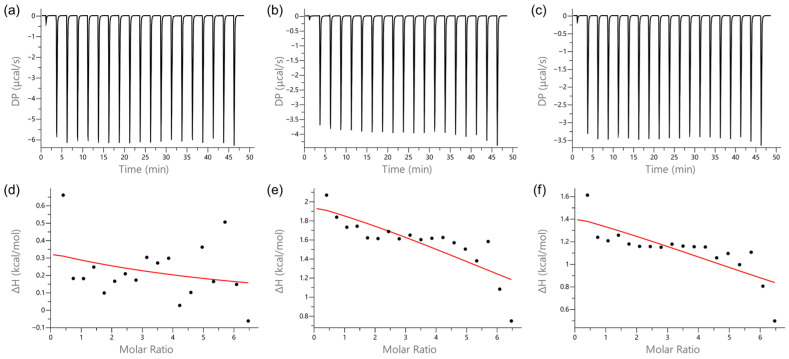
Representative ITC thermograms (upper panels) and corresponding binding isotherms (lower panels) for the interaction of the apoprotein ApoTi-CP with oxacillin (**a**,**d**), tetracycline (**b**,**e**), and streptomycin (**c**,**f**). Experiments were performed at 25 °C in TBS buffer using a MicroCal PEAQ-ITC instrument. ApoTi-CP (66 µM) was placed in the sample cell, and antibiotics (2 mM) were injected in 19 consecutive 2 µL injections at 150 s intervals under constant stirring (750 rpm). Raw heat signals are presented as differential power (DP) versus time, and the integrated heats (after subtraction of dilution controls) are plotted against the molar ratio of ligand to protein. Data were analyzed using the single set of sites binding. ApoTi-CP shows no detectable interaction with oxacillin, while only weak and poorly defined binding profiles are observed for tetracycline and streptomycin. The absence of well-defined saturation behavior and the low signal amplitude indicate marginal or non-specific interactions under the tested conditions.

**Figure 7:**
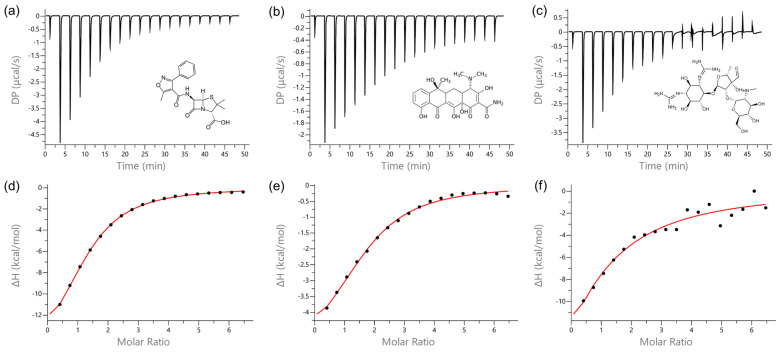
Representative ITC thermograms (upper panels) and corresponding binding isotherms (lower panels) for the interaction of native Ti-CP with oxacillin (**a**,**d**), tetracycline (**b**,**e**), and streptomycin (**c**,**f**). Experiments were performed at 25 °C in TBS buffer using a MicroCal PEAQ-ITC instrument. Ti-CP (66 µM) was placed in the sample cell, and antibiotics (2 mM) were titrated through 19 sequential injections of 2 µL at 150 s intervals under constant stirring (750 rpm). Raw heat signals are presented as differential power (DP) versus time, and integrated heats (after subtraction of dilution controls) are plotted against the molar ratio of ligand to protein. Binding isotherms were fitted using a single set of sites model. All detected interactions between Ti-CP and the tested antibiotics were exothermic. Oxacillin and tetracycline displayed moderate binding affinities with well-defined titration profiles, whereas streptomycin exhibited weaker and less well-defined binding behavior, consistent with lower affinity and reduced interaction specificity under the tested conditions.

ITC experiments revealed that Ti-CP and ApoTi-CP exhibit markedly different interaction profiles with oxacillin, tetracycline, and streptomycin (Figure 6 and Figure 7).

When oxacillin was titrated, no detectable interaction was observed for ApoTi-CP (Figure 6a), whereas Ti-CP showed clear binding to the antibiotic (Figure 7a). The interaction was characterized by a dissociation constant (*Kd*) of 33.7 × 10^−6^ ± 1.68 × 10^−6^ M, indicating moderate affinity. The binding process was exothermic, with a negative enthalpy change (*ΔH*) of −17.5 ± 0.469 kcal·mol^−1^, reflecting heat release upon complex formation. The binding stoichiometry (n) was estimated at 1.25 ± 1.8 × 10^−2^, suggesting that, on average, approximately five antibiotic molecules associate with four protein molecules.

A similar interaction pattern was observed for tetracycline. A poorly defined binding profile was detected for ApoTi-CP (Figure 6b), whereas Ti-CP interacted with tetracycline with a *Kd* of 39.0 × 10^−6^ ± 7.59 × 10^−6^ M (Figure 7b). The interaction was also exothermic, presenting a *ΔH* of −5.73 ± 0.530 kcal·mol^−1^. The estimated stoichiometry (*n* = 1.64 ± 6.8 × 10^−2^) indicates that roughly eight antibiotic molecules bind to every five protein molecules, suggesting the presence of multiple accessible interaction sites.

For streptomycin, no measurable interaction was detected with ApoTi-CP, but a weak interaction thermogram can be observed (Figure 6c). In contrast, Ti-CP exhibited a detectable but weaker interaction, with a *Kd* of 164 × 10^−6^ ± 30.7 × 10^−6^ M, indicating low affinity and higher variability in the binding parameters (Figure 7c). The interaction was strongly exothermic, with a *ΔH* of −57.5 ± 276 kcal·mol^−1^, consistent with substantial heat release during complex formation. The binding stoichiometry (*n* ≈ 0.6) suggests that, on average, one molecule of streptomycin interacts with every two protein molecules.

Quantitative thermodynamic parameters are summarized in Table 1.

Thermodynamic signature plots (*ΔG*, *ΔH* and −T*ΔS*) revealed that antibiotic binding to Ti-CP is predominantly enthalpy-driven, with ligand-dependent entropy penalties (Figure S3). Oxacillin exhibits favorable binding driven by a large negative enthalpy change, partially offset by a positive −T*ΔS* term, indicating an unfavorable entropy contribution. This pattern is consistent with the formation of stabilizing non-covalent interactions accompanied by conformational restriction and/or solvent ordering upon complex formation.

In contrast, tetracycline displays a more balanced thermodynamic profile, with favorable enthalpy and only minimal entropic penalty, resulting in efficient binding with limited conformational or solvent reorganization.

Streptomycin shows the most pronounced enthalpy–entropy compensation, characterized by a strongly favorable enthalpic contribution that is largely counterbalanced by a very large positive −T*ΔS* term. This indicates substantial entropic cost during binding, likely reflecting extensive structural organization and reduced conformational freedom associated with accommodation of a larger and highly polar ligand.

### 2.8. Antibacterial Effect and Combined Effects with Conventional Antibiotics

Although neither Ti-CP nor ApoTi-CP completely inhibited bacterial growth at any of the tested concentrations, both proteins interfered with bacterial growth in a significant manner, reducing growth relative to the untreated controls. Based on these observations, a fixed sub-inhibitory concentration of 125 µg·mL^−1^ was adopted for the antibiotic combination assays in order to assess potential modulatory effects of the proteins.

Effects of Ti-CP and ApoTi-CP combined with antibiotics are summarized in Table 2. In combination with oxacillin, both Ti-CP and ApoTi-CP exhibited additive effects against *S. aureus* ATCC 25923. Against the methicillin-resistant *S. aureus* (MRSA), Apo Ti-CP showed a synergistic interaction, reducing the MIC of oxacillin by fourfold, whereas Ti-CP displayed an additive effect. For *E. coli* ATCC 11303, both protein forms showed antagonistic interactions with oxacillin.

Tetracycline exhibited consistent synergistic interactions when combined with both protein across all bacterial strains tested, resulting in fourfold reductions in MIC values. This effect was observed for both Gram-positive and Gram-negative bacteria, indicating a broad potentiation of tetracycline activity by the protein.

Distinct interaction profiles were observed with streptomycin. Ti-CP showed synergistic effects against *E. coli* ATCC 11303 and *S. aureus* ATCC 700698, while no interaction was detected against *S. aureus* ATCC 25923. In contrast, ApoTi-CP exhibited additive effects against all tested strains when combined with streptomycin.

Detailed data on the results are presented in the Supplementary Materials (Tables S1 and S2).

### 2.9. Effects on Biofilm Formation and Bacterial Viability

**Figure 8:**
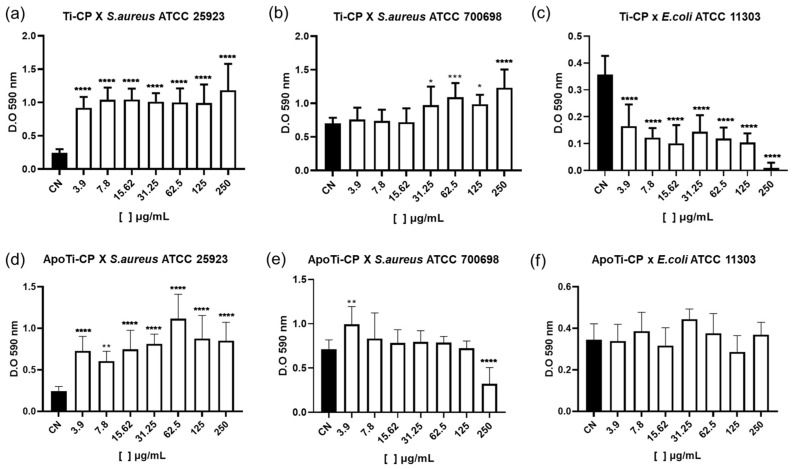
Effect of Ti-CP and ApoTi-CP on biofilm biomass formation. The impact of Ti-CP (**a**–**c**) and ApoTi-CP (**d**–**f**) on biofilm biomass was evaluated against *S. aureus* ATCC 25923, *S. aureus* ATCC 700698 (MRSA), and *E. coli* ATCC 11303. Biofilms were allowed to form in the presence of increasing protein concentrations (3.9–250 µg/mL), and total biomass was quantified by crystal violet staining, measured as absorbance at 590 nm (OD_590_). Results are presented as mean ± standard deviation. Statistical analysis was performed using one-way ANOVA followed by the appropriate post hoc test. Statistical significance relative to the untreated control (CN) is indicated as *p* < 0.05 (*), *p* < 0.01 (**), *p* < 0.001 (***), and *p* < 0.0001 (****).

**Figure 9:**
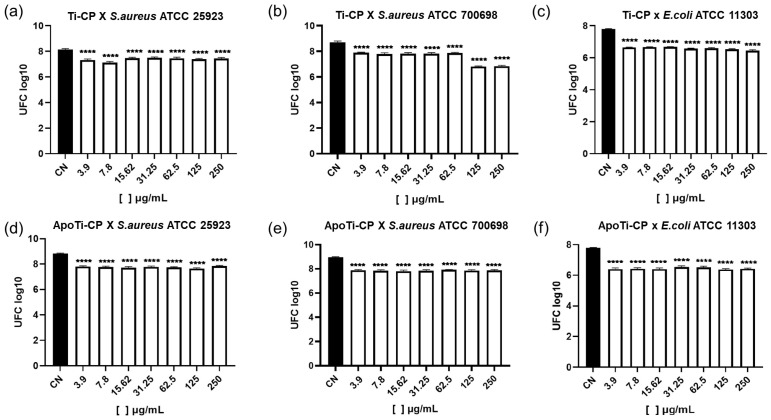
Effect of Ti-CP and ApoTi-CP on preformed bacterial biofilms evaluated by viable cell counts (CFU). The antibiofilm activity of Ti-CP (**a**–**c**) and ApoTi-CP (**d**–**f**) was assessed against biofilms formed by *S. aureus* ATCC 25923, *S. aureus* ATCC 700698 (MRSA), and *E. coli* ATCC 11303. After biofilm establishment, samples were treated with increasing protein concentrations (3.9–250 µg/mL), and biofilm viability was quantified by counting colony-forming units (CFU). Results are expressed as mean ± standard deviation. Statistical significance relative to the untreated control (CN) is indicated as *p* < 0.0001 (****).

Neither Ti-CP nor ApoTi-CP significantly reduced the total biofilm biomass formed by *S. aureus* ATCC 25923 across the concentration range tested (Figure 8a,d). However, both proteins promoted a significant reduction in viable cells within the biofilm, with an average decrease of approximately one logarithmic unit relative to the control, indicating a marked effect on bacterial viability despite limited impact on biofilm mass (Figure 9a,d).

For the methicillin-resistant strain *S. aureus* ATCC 700698, ApoTi-CP significantly reduced biofilm biomass at the highest concentration tested (250 μg·mL^−1^), reaching approximately 50% inhibition (Figure 8b,e). Both Ti-CP and ApoTi-CP significantly decreased viable cell counts at all concentrations, with Ti-CP displaying a stronger effect at higher concentrations (125–250 μg·mL^−1^), corresponding to a reduction of up to 1.8 log units (Figure 9b–e).

In *E. coli* ATCC 11303, Ti-CP exhibited significant antibiofilm activity, reducing biofilm biomass by approximately 60% at all tested concentrations and completely inhibiting biofilm formation at 250 μg·mL^−1^. ApoTi-CP caused only a modest, non-significant reduction in biofilm biomass (Figure 8c,f). Notably, both proteins significantly reduced the number of viable cells, with decreases of approximately two logarithmic units relative to the untreated control (Figure 9c,f).

### 2.10. Cytotoxicity Assessment

The cytotoxicity profile of Ti-CP was evaluated in murine L929 fibroblasts using the MTT assay after 48 h of exposure (Figure S3). At concentrations ranging from 7.8 to 62.5 µg·mL^−1^, no significant reduction in cell viability was observed relative to the untreated control (*p* > 0.05), with viability values remaining close to or slightly above 100%. At 125 µg·mL^−1^, a statistically significant reduction in viability was detected; however, cell viability remained above 50%.

In contrast, the two highest concentrations tested (250 and 500 µg·mL^−1^) resulted in marked cytotoxic effects, with a drastic reduction in cell viability (*p* < 0.01 and *p* < 0.0001, respectively). These findings indicate that Ti-CP exhibits concentration-dependent cytotoxicity, with a favorable in vitro biocompatibility profile at lower concentrations.

## 3. Discussion

Antimicrobial resistance (AMR) is recognized as one of the most pressing global public health threats of the twenty-first century. Current estimates indicate that bacterial AMR was directly responsible for more than 1.27 million deaths in 2019 and contributed to nearly 5 million deaths globally when associated mortality is considered [16]. Without effective intervention, these burdens are projected to increase substantially over the coming decades, with long-term modelling studies forecasting a steady rise in deaths attributable to resistant infections, amounting to tens of millions by mid-century [16,17]. This escalating crisis undermines the efficacy of existing antibiotics, compromises routine clinical care, and emphasizes the urgent need for innovative therapeutic strategies. In this context, approaches that combine conventional antibiotics with adjunctive molecules to potentiate their activity have gained considerable attention as potential means to extend antibiotic effectiveness and circumvent resistance mechanisms [18,19,20].

The present study was designed within this conceptual framework to evaluate the biophysical properties of a novel marine sponge carotenoprotein and to explore its ability, and that of its pigment-depleted form, to modulate antibiotic interactions and antimicrobial activity.

Carotenoproteins have been described in a limited number of biological systems and remain comparatively underexplored [21]. Classical examples include β-crustacyanin from crustaceans, which stabilizes AXT dimers and induces pronounced bathochromic shifts in absorption [8,22], the orange carotenoid protein (OCP) from cyanobacteria, which plays a central role in photoprotection through light-induced conformational switching [4], and ERPs, which bind hydrophobic ligands and have been implicated in extracellular matrix interactions and neural plasticity [9,10,14].

Despite major differences in biological function, these systems share fundamental structural principles: carotenoids are stabilized within hydrophobic protein pockets, enabling their solubilization in aqueous environments and often modulating both protein conformation and function. Carotenoid–protein complexes across diverse taxa serve roles ranging from coloration and photoprotection to molecular transport, highlighting their functional versatility despite limited structural homology between families. Ti-CP appears to share the general structural paradigm of carotenoid-mediated stabilization and functional modulation, while differing in its supramolecular behavior and interaction profile, suggesting that sponge carotenoproteins may represent a distinct functional adaptation within the expanding diversity of carotenoid-binding proteins.

Carotenoproteins from marine sponges remain poorly documented, with only a few isolated reports addressing their biochemical properties [9,10,23,24]. In this context, Ti-CP represents a novel addition to this limited repertoire, distinguished by its clear separation into holo- and apo-forms, its β-sheet-rich secondary structure, and its pronounced dependence on the carotenoid moiety for structural stability and functional behavior.

Like carotenoproteins described from *Haliclona* species [9,10], Ti-CP accommodates two carotenoid molecules per protein unit, reinforcing a conserved structural theme among sponge-derived carotenoproteins. Our results suggest that these pigments correspond to AXT and MXT, mirroring the carotenoid composition previously reported for *Haliclona* sp. carotenoproteins [9,10]. However, assignments shown in Ti-CP should be considered putative, as confirmatory structural characterization using authentic standards or MS/MS fragmentation was not performed.

The dual-carotenoid arrangement has also been observed in other sponge proteins, such as the lectin from *Haliclona caerulea*, which binds AXT, and the lectin from *Haliclona manglaris*, which interacts with both AXT and MXT [11,13]. In *Haliclona* sp., structural analyses have suggested that both carotenoids are accommodated within a single, wide hydrophobic pocket, rather than in independent binding sites [9,10].

Consistent with this model, removal of the pigment from Ti-CP resulted in a visually evident transition from a blue native complex to a colorless apoprotein and an orange carotenoid fraction, reflecting a pronounced shift in the electronic environment of the chromophore and a corresponding change in visible absorption, similar behavior was observed in *H. caerulea* [11]. Protein-induced spectral shifts in carotenoids are commonly attributed to changes in the electronic structure of the polyene chain caused by protein–chromophore interactions, including conformational distortion, altered polarizability, and excitonic coupling, as extensively described for crustacyanin and the orange carotenoid protein [25].

Although pigment removal did not induce global secondary structure unfolding, the pronounced increase in polydispersity and aggregation propensity observed for ApoTi-CP suggest that the carotenoid plays a structural role beyond simple chromophore association. Thus, the pigment may contribute to stabilizing the protein’s supramolecular organization in solution, likely by modulating intermolecular interactions and reducing aggregation-prone conformational states. Such behavior is consistent with the notion that carotenoids can act as structural cofactors in carotenoproteins, helping maintain conformational integrity at higher-order assembly levels rather than stabilizing local secondary structure.

ApoTi-CP was initially hypothesized to function as a potential drug carrier, based on the assumption that hydrophobic regions would remain accessible after pigment removal. However, the absence of measurable binding to antibiotics indicates that carotenoid depletion compromises the protein’s capacity to establish stable ligand interactions. This loss of binding competence is consistent with the structural perturbations inferred from DLS.

In contrast, Ti-CP displayed reproducible interactions with all tested antibiotics, indicating that the intact pigment–protein assembly is necessary to preserve a functional binding interface. These observations support the view that the carotenoid is not merely a passive chromophore but an integral structural component that contributes to the formation or stabilization of ligand-accessible domains.

Therefore, we propose that the hydrophobic pocket that accommodates the carotenoid in Ti-CP is sufficiently large and structurally organized to allow the transient binding of other hydrophobic molecules. The binding affinities followed a size-dependent trend, with oxacillin showing the strongest interaction, followed by tetracycline, while the bulkier streptomycin interacted weakly. This hierarchy supports the notion that steric accessibility within the binding pocket constrains ligand accommodation. In fact, thermodynamic signatures indicate that while binding is consistently driven by favorable interaction energetics, the magnitude of entropy compensation varies substantially among ligands, reflecting differences in molecular size, polarity, and structural adaptability within the Ti-CP binding environment.

The thermogram signals observed for streptomycin and tetracycline in the ApoTi-CP, despite the absence of calculable binding parameters, further suggest nonspecific or transient interactions rather than a defined binding mode. Interestingly, this functional behavior finds parallels in ERPs, which are evolutionarily linked to carotenoproteins yet do not bind endogenous carotenoids in vertebrates; instead, they have been implicated in the trafficking of hydrophobic molecules, such as lipids, within lysosomal compartments [14,15].

When the combined effects of Ti-CP and ApoTi-CP are interpreted in light of the ITC data, it becomes evident that antibiotic potentiation cannot be attributed to a single, clearly defined molecular mechanism. Although binding was detected for Ti-CP, the microbiological outcomes suggest that additional indirect biological interactions may be involved. At present, the available data do not allow discrimination between direct protein–drug interactions and broader effects on bacterial physiology. Therefore, the interpretations proposed here should be regarded as plausible hypotheses rather than experimentally demonstrated mechanisms.

For oxacillin, both protein forms produced largely similar outcomes across strains, including antagonism against *E. coli*. This discrepancy argues against a model in which the ApoTi-CP acts as a carrier and suggests that the biological outcome is driven by interactions occurring at the cell level rather than by stable protein–drug complexation. In Gram-negative bacteria, oxacillin is intrinsically limited by outer-membrane permeability and by β-lactam resistance determinants [26,27]; thus, any protein-induced sequestration of the drug, changes in diffusion within the medium, or stabilization of the antibiotic away from its periplasmic targets could plausibly manifest as antagonism.

In contrast, for tetracycline, both (Ti-CP and ApoTi-CP) consistently showed synergy across all strains, even though only Ti-CP exhibited defined binding in ITC. Because tetracycline activity depends on intracellular accumulation and is strongly affected by permeability and efflux [28,29], the uniform potentiation observed with tetracycline may reflect indirect modulation of bacterial physiology, potentially influencing effective intracellular antibiotic exposure. However, this possibility was not directly evaluated in the present study and remains to be experimentally confirmed. Overall, the mismatch between ITC binding and microbiological outcomes indicates that, while carotenoid-dependent organization enables direct ligand binding in Ti-CP, the dominant drivers of synergy are likely strain- and drug-dependent biological interactions rather than simple transport of antibiotics by ApoTi-CP.

The most informative contrasts emerged with streptomycin, where distinct interaction profiles depending on the strain background were observed. Streptomycin requires active transport and membrane potential for uptake and can be compromised by permeability barriers, biofilm physiology, and aminoglycoside-modifying enzymes [30,31]. The weak, poorly defined calorimetric signal observed for ApoTi-CP with streptomycin suggests that any interaction is transient or nonspecific, yet biologically the protein still produced additive effects in multiple contexts, whereas Ti-CP displayed synergy in resistant *S. aureus* and in *E. coli*. This pattern is compatible with the possibility that Ti-CP influences streptomycin performance through a combination of structural organization and broader physiological effects.

Protein-based antibiotic adjuvants are increasingly reported in the literature, including antimicrobial peptides, enzymes, and lectins, which can potentiate antibiotics by perturbing membranes, interfering with cell wall integrity, thereby enhancing drug entry or reducing efflux [32,33,34]. In sponge-derived systems, lectins in particular have been proposed to interact with cell-surface glycans and compromise membrane-associated processes, favoring antibiotic susceptibility [35,36].

In the case of carotenoproteins, the mechanistic space remains less defined; however, their carotenoid components and pigment–protein architecture are frequently associated with redox buffering and antioxidant behavior [5,13], which could indirectly influence bacterial stress responses and biofilm physiology. In addition, certain carotenoids have been reported to interfere with biofilm-associated phenotypes. For instance, the carotenoid crocetin not only displays antibiofilm activity against staphylococcal strains but also significantly enhances the antibiofilm efficacy of tobramycin when used in combination, with combined treatments achieving markedly greater biofilm reduction than either agent alone [37]. Overall, the observed strain- and drug-dependent modulation of antibiotic activity highlights Ti-CP and ApoTi-CP as biologically active systems worthy of further investigation. However, additional mechanistic and in vivo studies will be necessary to determine their precise mode of action and translational potential.

## 4. Materials and Methods

### 4.1. Sponge Collection and Extraction

Specimens of the marine sponge *Tedania ignis* were manually collected in the intertidal zone of Bitupitá beach (Barroquinha, Ceará, Brazil). Samples were transported on ice and stored at −20 °C until processing. Sponge tissue was homogenized in distilled water (1:2, *w*/*v*), filtered through nylon mesh, and centrifuged (9000× *g*, 20 min, 4 °C). The supernatant was collected as the crude aqueous extract.

A voucher specimen was deposited in the Collection of the Marine Invertebrates of the Federal University of Ceará (UFC) under accession number POR-419. Sampling was conducted under authorization from SISBIO (permit no. 33913-13), and access to the sponge genetic heritage was registered in SISGEN under code A1792FE.

### 4.2. Protein Purification

Proteins were fractionated by ammonium sulfate (Sigma-Aldrich, St. Louis, MO, USA) precipitation (0–30%, 30–60%, and 60–90% saturation). The 60–90% fraction was solubilized in 50 mM Tris-HCl buffer, pH 7.6 (TBS; Sigma-Aldrich, St. Louis, MO, USA), dialyzed against distilled water, and lyophilized. The carotenoprotein (Ti-CP) was purified by anion-exchange chromatography (HiTrap Q FF, ÄKTA Pure, Cytiva, Marlborough, MA, USA). The column was equilibrated and washed with TBS. Adsorbed proteins were eluted using a linear gradient of 0–1 M NaCl (Sigma-Aldrich, St. Louis, MO, USA) in the equilibration buffer. Blue color-containing fractions were pooled, dialyzed, and lyophilized.

### 4.3. Assessment of Purity and Molecular Mass

Protein purity was evaluated by SDS-PAGE (15%) under reducing and non-reducing conditions [38]. Low Molecular Weight (LMW) marker kit (Sigma-Aldrich, Saint Louis, MO, USA) was used as the migration standard to estimate the apparent molecular mass of the protein.

Native molecular mass was estimated by SEC using a BioSuite SEC HR 250 (5 µm, 7.8 × 300 mm) column coupled to an ACQUITY H-Class UPLC system (Waters Corp., Milford, MA, USA), calibrated with molecular-mass standards (Sigma Aldrich, Saint Louis, MO, USA). The column was equilibrated and eluted with TBS at a flow rate of 0.3 mL·min^−1^. Protein elution was monitored using PDA at 280 nm and across the visible range (400–700 nm) to simultaneously track protein and pigment-containing species. The degree of protein purity was also quantified using Empower 3 software (Waters Corp., Milford, MA, USA, version 3.9.0) based on chromatographic peak integration at 280 nm.

### 4.4. Pigment Removal and Reverse-Phase Chromatography

Ti-CP was separated into apoprotein and pigment by RPC using a BEH C8 column (1.7 µm, 2.1 × 100 mm) coupled to an ACQUITY H-Class UPLC system Waters Corp., Milford, MA, USA). The column was equilibrated in 0.1% trifluoroacetic acid (TFA; Sigma-Aldrich, St. Louis, MO, USA) containing 5% acetonitrile (ACN; Sigma-Aldrich, St. Louis, MO, USA), and retained components were eluted with a linear gradient of ACN from 5 to 70% in 0.1% TFA. Eluted fractions were collected and dried under vacuum.

Protein-containing fractions (ApoTi-CP) were stored for subsequent analyses, whereas pigment-containing fractions were resuspended in acetone (Sigma-Aldrich, St. Louis, MO, USA) and subjected to a second reverse-phase separation on a BEH C18 column (1.7 µm, 2.1 × 50 mm). The column was equilibrated with methanol (acetone (Sigma-Aldrich, St. Louis, MO, USA)/water (80:20, *v*/*v*; solvent A) and eluted with a linear gradient of acetone/methanol (1:1, *v*/*v*; solvent B) at a flow rate of 1.0 mL·min^−1^, allowing further resolution of the carotenoid species. Elution was monitored using a PDA.

### 4.5. Circular Dichroism Spectroscopy

Far-UV CD spectra of Ti-CP and ApoTi-CP were recorded on a Jasco J-815 spectropolarimeter (Jasco International Co., Tokyo, Japan) equipped with a Peltier temperature-control system. Protein samples were prepared in TBS and measured in a 1 mm path-length quartz cuvette. Spectra were acquired in the range of 190–240 nm at 20 °C. Each spectrum was baseline-corrected and averaged from four repeated scans. Secondary structure content was estimated using the BestSel online server [39].

The thermal stability of the secondary structure of Ti-CP was evaluated by monitoring temperature-induced unfolding. Protein samples were subjected to a temperature ramp from 25 to 100 °C (3 °C/min) while ellipticity was continuously monitored at 215 nm. The melting temperature (Tm) was determined using the Thermal Denaturation tool available on the BestSel online server [39].

### 4.6. Hydrodynamic Size Distributions

DLS measurements were performed using a Zetasizer Advance Ultra (Malvern Panalytica, Worcestershire, UK) at 20 °C. Ti-CP and ApoTi-CP were prepared in ultrapure water at final concentrations of 250 µg·mL^−1^. Prior to analysis, samples were centrifuged at 10,000× *g* for 20 min at 4 °C to remove insoluble material and dust particles. Supernatants were transferred to clean disposable polystyrene cuvettes to avoid contamination or air bubble formation.

Size distributions were calculated from the autocorrelation functions using the general-purpose analysis model provided by the manufacturer, generating intensity-, volume-, and number-weighted hydrodynamic diameter distributions. Each condition was measured in triplicate, and each reported measurement represents the average of multiple scans. Experimental procedures were adapted from [40], with minor modifications to accommodate buffer variation.

### 4.7. Binding of Antibiotics to Ti-CP and ApoTi-CP

ITC experiments were performed using a MicroCal PEAQ-ITC Analysis software (Malvern Panalytica, Worcestershire, UK), version 1.41, equipped with a 250 µL sample cell and a 40 µL injection syringe. Interactions between Ti-CP or ApoTi-CP and the antibiotics tetracycline, oxacillin, and streptomycin were analyzed in TBS. All experiments were conducted at 25 °C, and the reference cell was filled with distilled water.

Protein solutions were adjusted to 66 µM and placed in the sample cell, while antibiotics were prepared at 2 mM in the same buffer and loaded into the syringe. Prior to titration, all solutions were thoroughly degassed to prevent bubble formation. Titrations were performed as 19 consecutive injections of 2 µL at 150 s intervals under constant stirring (750 rpm).

Control titrations of each antibiotic into buffer were performed under identical conditions to account for dilution heats, and these values were subtracted from the corresponding binding isotherms. Integrated heat data were fitted by nonlinear regression using the single set of sites binding model implemented in the MicroCal PEAQ-ITC Analysis software (Malvern Panalytica, Worcestershire, UK), version 1.41. The fitting procedure yielded the dissociation constant (*Kd*), binding enthalpy (*ΔH*), and stoichiometry (*n*). Thermodynamic parameters, including Gibbs free energy (*ΔG*) and entropy contribution (−T*ΔS*), were calculated by the software, providing the full thermodynamic signature of the interaction.

### 4.8. Antibacterial, Antibiofilm and Antibiotic Combination Assays

#### 4.8.1. Bacterial Strains and Culture Conditions

The antibacterial and antibiofilm assays were performed using *Staphylococcus aureus* ATCC 25923, *S. aureus* ATCC 700698 (MRSA), and *Escherichia coli* ATCC 11303. Bacterial strains were maintained on Mueller–Hinton agar (MHA; HiMedia Laboratories, Mumbai, India) plates and incubated at 37 °C for 24 h. Single colonies were transferred to Mueller–Hinton broth (MHB; HiMedia Laboratories, Mumbai, India) and incubated under the same conditions. Cells were harvested by centrifugation (9000× *g*, 5 min, 4 °C), resuspended in fresh MHB, and the cell density was adjusted to 5 × 10^5^ CFU·mL^−1^ based on optical density at 620 nm.

#### 4.8.2. Antibacterial Assay and Combination Between Proteins and Antibiotics (Checkerboard Assay)

Antibacterial activity was evaluated using the broth microdilution method according to the Clinical and Laboratory Standards Institute (CLSI) guidelines—M07-A10, with minor modifications. Bacterial suspensions were adjusted to 5 × 10^5^ CFU·mL^−1^ in Mueller–Hinton Broth (MHB) and inoculated into 96-well microplates containing Ti-CP or ApoTi-CP solutions at 3.9–250 μg·mL^−1^; or antibiotics (tetracycline, oxacillin, and streptomycin). Plates were incubated at 37 °C for 24 h under static conditions. Growth and sterility controls were included in every plate. The minimum inhibitory concentration (MIC) was defined as the lowest concentration of protein at which no visible bacterial growth was detected following incubation.

Combined effect between Ti-CP or ApoTi-CP and the antibiotics tetracycline, oxacillin, and streptomycin was evaluated using the checkerboard microdilution method. Antibiotics were serially diluted vertically in microplates at concentrations corresponding to MIC, 1/2 × MIC, 1/4 × MIC, 1/8 × MIC, and 1/16 × MIC, while Ti-CP or ApoTi-CP was added horizontally at a constant concentration of 125 µg·mL^−1^. Each well contained 50 µL of antibiotic solution, 50 µL of protein solution, and 100 µL of bacterial inoculum (5 × 10^5^ CFU·mL^−1^).

Plates were incubated at 37 °C for 24 h, and bacterial growth was quantified by measuring optical density at 620 nm. Control wells included antibiotics alone (MIC control) and growth controls without antimicrobials. Interactions were classified as synergistic (1/4–1/16 × MIC), additive (1/2 × MIC), indifferent (1 × MIC), or antagonistic (≥2 × MIC), based on the change in MIC values in the presence of the proteins [35].

#### 4.8.3. Biofilm Formation Assay

The effect of Ti-CP and ApoTi-CP on bacterial biofilm formation was evaluated in 96-well polystyrene microplates using tryptone soy broth (TSB; HiMedia Laboratories, Mumbai, India), according to [41], with modifications. Protein solutions were prepared at concentrations ranging from 3.9 to 250 µg·mL^−1^ and mixed with 100 µL of bacterial suspension adjusted to 1 × 10^6^ cells·mL^−1^ in each well. Plates were incubated at 37 °C for 24 h under static conditions to allow biofilm formation. Growth and sterility controls were included in every plate. Bacterial growth in the planktonic phase was monitored by measuring the optical density at 620 nm using a SpectraMax i3 microplate reader (SpectraMax^®^ i3, Molecular Devices, Sunnyvale, CA, USA).

#### 4.8.4. Quantification of Biofilm Biomass

Total biofilm biomass was quantified using the crystal violet (CV; Sigma-Aldrich, St. Louis, MO, USA) staining method. After incubation, the culture medium was removed, and the wells were gently washed twice with 200 µL of sterile distilled water to remove loosely attached cells. Biofilms were fixed with 200 µL of 99% methanol (Sigma-Aldrich, St. Louis, MO, USA) for 15 min, air-dried, and stained with 200 µL of crystal violet solution for 5 min. Excess dye was removed by washing with water, and the bound stain was solubilized with 200 µL of 33% (*v*/*v*) acetic acid (Sigma-Aldrich, St. Louis, MO, USA). The optical density was measured at 590 nm (OD590) using a microplate reader (SpectraMax^®^ i3, Molecular Devices, Sunnyvale, CA, USA), which is proportional to the total biofilm biomass.

#### 4.8.5. Quantification of Viable Biofilm Cells

To determine the number of viable cells embedded in the biofilm, wells were washed twice with sterile distilled water and then filled with 200 µL of water. Plates were subjected to ultrasonic bath treatment for 8 min to disrupt the biofilm matrix and release bacterial cells. The resulting suspensions were serially diluted and plated on tryptone soy agar (TSA; HiMedia Laboratories, Mumbai, India). After incubation at 37 °C for 24 h, colonies were counted and expressed as log CFU·mL^−1^.

#### 4.8.6. Cell Viability Assay

The cytotoxicity of Ti-CP was evaluated using the murine fibroblast cell line L929 through the colorimetric MTT assay (3-(4,5-dimethylthiazol-2-yl)-2,5-diphenyltetrazolium bromide). L929 cells were cultured in Dulbecco’s Modified Eagle Medium (DMEM; Gibco, Thermo Fisher Scientific, Waltham, MA, USA) supplemented with 10% fetal bovine serum (FBS; Gibco, Thermo Fisher Scientific, Waltham, MA, USA) under standard conditions (37 °C, 5% CO_2_). Cells were seeded in 96-well flat-bottom plates at a density of 5 × 10^4^ cells per well and allowed to adhere overnight. After adhesion, cells were exposed to Ti-CP at concentrations ranging from 7.8 to 500 µg·mL^−1^ for 48 h. Control cells were maintained under identical conditions without protein treatment. Following the exposure period, 100 µL of MTT solution (Sigma-Aldrich, St. Louis, MO, USA) was added to each well and plates were incubated for 4 h. The resulting formazan crystals were solubilized, and the absorbance was measured at 570 nm using a microplate reader (SpectraMax^®^ i3, Molecular Devices, Sunnyvale, CA, USA).

Cell viability (%) was calculated using the following equation:

$$Cell\;viability\;\left(\%\right)=\;\frac{{\bar{Abs}}_{570\;nm}(Ti-CP)}{{\bar{Abs}}_{570\;nm}\;(control)}\times 100\%$$

#### 4.8.7. Statistical Analysis

All experiments were performed in at least three independent biological replicates, and each measurement was conducted in technical triplicates unless otherwise stated. Data are presented as mean ± standard deviation (SD). Statistical analyses were performed using GraphPad Prism (version 9.0, GraphPad Software, San Diego, CA, USA). Differences among multiple groups were evaluated by one-way analysis of variance (ANOVA) followed by Bonferroni’s post hoc test for pairwise comparisons. Treatment groups were compared to the corresponding untreated controls. The value of *p* < 0.05 was considered statistically significant.

In the biofilm and viability assays, each treatment concentration was compared specifically with the untreated control, and *p* values were adjusted accordingly to control for multiple comparisons. For cytotoxicity analysis (MTT assay in L929 fibroblasts), ANOVA was followed by Tukey’s post hoc test for multiple comparisons. The value of *p* < 0.05 was considered statistically significant.

## 5. Conclusions

This work aimed to evaluate a sponge-derived carotenoprotein as a potential antibiotic carrier through its apoprotein form. However, pigment removal resulted in loss of supramolecular stability and prevented defined interactions with antibiotics, indicating that ApoTi-CP is unlikely to function as an efficient delivery scaffold through specific binding mechanisms. In contrast, Ti-CP was structurally stable, interacted with antibiotics, and modulated antibacterial and antibiofilm activities in a drug- and strain-dependent manner. The observed synergistic effects, particularly with tetracycline, suggest that antibiotic potentiation is largely mediated by indirect biological mechanisms rather than by stable protein–drug complexation. Although higher concentrations induced significant cytotoxicity, lower concentrations displayed acceptable in vitro biocompatibility, supporting continued investigation under controlled dosing conditions. These findings demonstrate that the structural state of Ti-CP influences its interaction profile with antibiotics and modulates antibacterial responses in vitro. While the underlying mechanisms remain to be clarified, the data provide a foundation for future studies aimed at understanding the functional interplay between sponge-derived carotenoproteins and conventional antibiotics.

## Figures and Tables

**Table 1:** Thermodynamic parameters obtained by ITC for the interaction between Ti-CP and antibiotics.

Antibiotic	*Kd* (µM)	*Ka* (M^−1^) (×10^4^)	*n* (Stoichiometry)	*ΔG* (kcal·mol^−1^)	R^2^ Value
Oxacillin	33.7 ± 1.68	3.0	1.25 ± 0.02	−6.07	0.787
Tetracycline	39.0 ± 7.59	2.6	1.64 ± 0.07	−5.94	0.490
Streptomycin	164 ± 307	0.61	~0.6	−5.19	0.454

Thermodynamic parameters were obtained using a one-site binding model. Values are presented as mean ± standard deviation. The association constant (*Ka*) was calculated as the inverse of the dissociation constant (*Kd*). The stoichiometry (*n*) represents the number of antibiotic molecules bound per Ti-CP molecule. The Gibbs free energy change (*ΔG*) was calculated according to the equation *ΔG* = −RT ln *Ka*, assuming a temperature of 298 K. For streptomycin, the high uncertainty in *Kd* reflects weak binding and limited fitting accuracy. R^2^ value represents the coefficient of determination obtained from nonlinear regression fitting of the binding isotherms using the one-set-of-sites model in MicroCal PEAQ-ITC Analysis software (v.1.41).

**Table 2:** Combined effects of Ti-CP and ApoTi-CP with antibiotics against bacterial strains.

Bacterial Strain	Antibiotic	Ti-CP	ApoTi-CP
*S. aureus* ATCC 25923	Oxacillin	Additive (1/2)	Additive (1/2)
*S. aureus* ATCC 25923	Tetracycline	Synergistic (1/4)	Synergistic (1/4)
*S. aureus* ATCC 25923	Streptomycin	No interaction (1)	Additive (1/2)
*S. aureus* ATCC 700698 (MRSA)	Oxacillin	Additive (1/2)	Synergistic (1/4)
*S. aureus* ATCC 700698 (MRSA)	Tetracycline	Synergistic (1/4)	Synergistic (1/4)
*S. aureus* ATCC 700698 (MRSA)	Streptomycin	Synergistic (1/4)	Additive (1/2)
*E. coli* ATCC 11303	Oxacillin	Antagonistic (>1)	Antagonistic (>1)
*E. coli* ATCC 11303	Tetracycline	Synergistic (1/4)	Synergistic (1/4)
*E. coli* ATCC 11303	Streptomycin	Synergistic (1/4)	Additive (1/2)

Values in parentheses indicate the fold reduction in the antibiotic minimum inhibitory concentration (MIC) after association with the protein (Ti-CP or ApoTi-CP at 125 µg·mL^−1^), relative to the MIC of the antibiotic alone. A value of 1 indicates no interaction, 1/2 indicates an additive effect, values of 1/4 or lower indicate synergistic interaction, and values greater than 1 (>1) indicate an antagonistic effect, corresponding to reduced antibiotic efficacy in the presence of the protein.

## Data Availability

The data presented in this study are available in the article and its Supplementary Materials. Further inquiries can be directed to the corresponding author.

## Supplementary Materials

The following supporting information can be downloaded at: https://www.mdpi.com/article/10.3390/md24030118/s1. Figure S1: Purification and characterization of the carotenoprotein Ti-CP from *Tedania ignis*; Figure S2: Size-exclusion chromatography (SEC) coupled to photodiode array detection (PDA) of Ti-CP; Figure S3. Thermal stability of Ti-CP monitored by CD; Figure S4. Thermodynamic signature plots for antibiotic binding to Ti-CP determined by ITC; Figure S5. Cytotoxicity assessment of Ti-CP in L929 murine fibroblasts after 48 h of exposure; Table S1: Combined effects of Ti-CP with antibiotics against bacterial strains; Table S2. Combined effects of ApoTi-CP with antibiotics against bacterial strains.

## Abbreviations

The following abbreviations are used in this manuscript:

AMR	Antimicrobial resistance
ANOVA	Analysis of variance
AXT	Astaxanthin
ApoTi-CP	Apocarotenoprotein from *Tedania ignis*
CD	Circular dichroism
CFU	Colony-forming units
CLSI	Clinical and Laboratory Standards Institute
CV	Crystal violet
DLS	Dynamic light scattering
DP	Differential power
ESI-MS	Electrospray ionization mass spectrometry
ITC	Isothermal titration calorimetry
Kd	Dissociation constant
Ka	Association constant
MIC	Minimum inhibitory concentration
MRSA	Methicillin-resistant *Staphylococcus aureus*
MXT	Mytiloxanthin
OD	Optical density
OCP	Orange carotenoid protein
PDA	Photodiode array detector
RPC	Reverse-phase chromatography
SEC	Size-exclusion chromatography
TBS	Tris-buffered saline
TFA	Trifluoroacetic acid
Ti-CP	Carotenoprotein from *Tedania ignis*
TSB	Tryptone soy broth
TSA	Tryptone soy agar
