# Supplementary material for: Biophysical Characterization of a Carotenoprotein from Marine Sponge Tedania ignis Reveals Pigment-Dependent Stability and Antibiotic Interactions
Source: Mar Drugs. 2026 Mar 21;24(3):118. doi: 10.3390/md24030118 (PMC13027469; doi:10.3390/md24030118)
Supplement: Supplementary file 1 [file marinedrugs-24-00118-s001.zip › marinedrugs-4162497-supplementary.pdf]

Supplementary Material – Manuscript: Biophysical Characterization of a Carotenoprotein from marine sponge *Tedania ignis* Reveals Pigment-Dependent Stability and Antibiotic Interactions

Philippe Lima Duarte[1], Paulo Anderson Paiva Martins[1], Jéssica de Assis Duarte[1], Manoel Ferreira da Costa Filho[1], Ellen Araújo Malveira[2], Celso Shiniti Nagano[1], Alexandre Holanda Sampaio[1], Edson Holanda Teixeira[2], Rômulo Farias Carneiro[1]*, and Mayron Alves de Vasconcelos[2,3]*

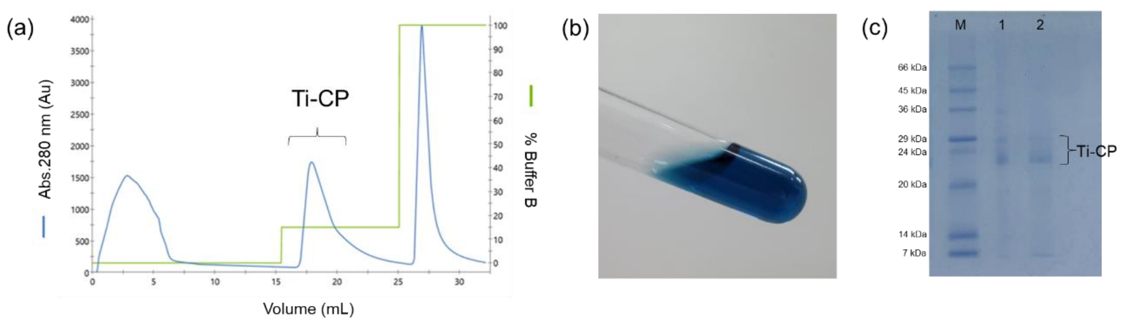

Figure S1. Purification and characterization of the carotenoprotein Ti-CP from *Tedania ignis*. (A) Anion-exchange chromatography profile of the Ti-CP-enriched fraction obtained after ammonium sulfate precipitation (60–90%). The column was equilibrated with buffer A (50 mM Tris-HCl, pH 7.6) and proteins were eluted using buffer B (50 mM Tris-HCl, pH 7.6, supplemented with 1 M NaCl). Protein elution was monitored at 280 nm (blue line), and the NaCl steps are shown in green. The Ti-CP-containing fractions eluted at approximately 150 mM NaCl and are indicated by the bracket. (B) Representative image of the purified Ti-CP fraction displaying an intense blue coloration, characteristic of pigment-bound carotenoproteins. (C) SDS-PAGE analysis of the purified fractions. Lane M: molecular mass marker; lane 1: enriched fraction; lane 2: Ti-CP, showing a broad protein band centered around ~25 kDa.

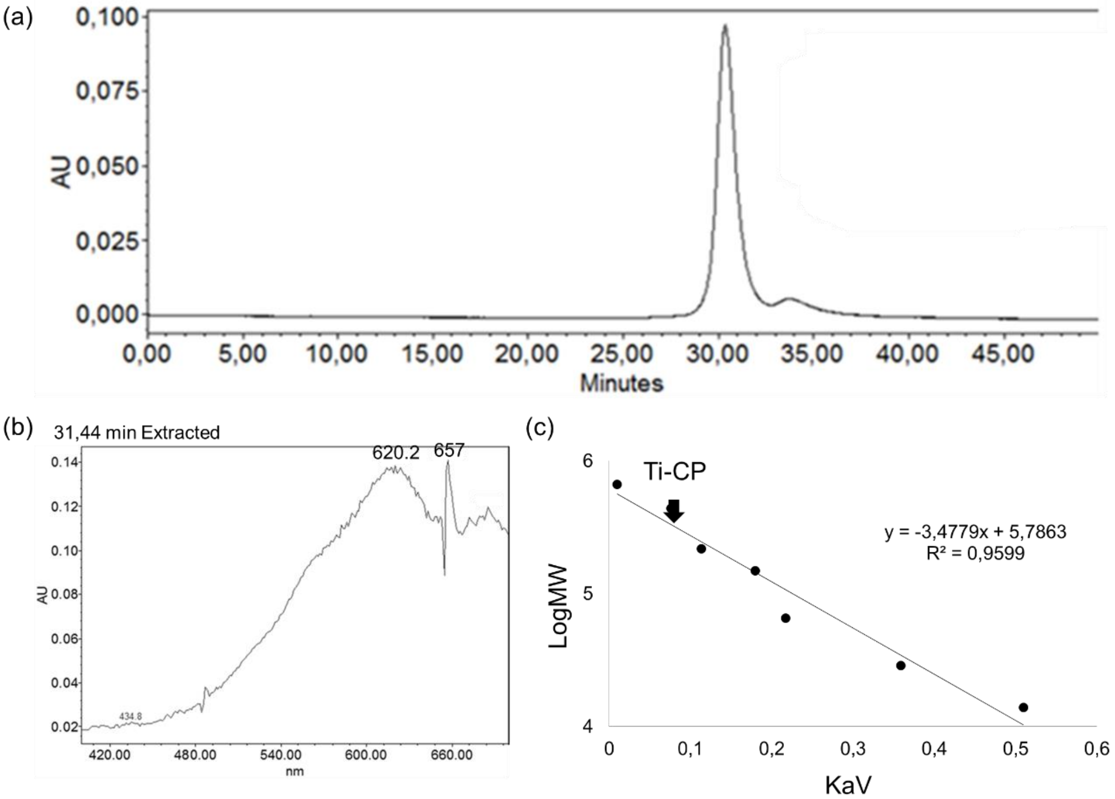

Figure S2. Chromatographic and spectroscopic characterization of Ti-CP. (A) Size-exclusion chromatography (SEC) profile of Ti-CP monitored at 280 nm, showing the main protein elution peak. (B) Visible-range photodiode array (PDA) absorption spectrum recorded from the eluted fraction, revealing the characteristic spectral features of the pigment–protein complex. (C) Calibration curve used for molecular mass estimation based on SEC elution behavior, showing the correlation between elution volume and the logarithm of molecular mass of standard proteins.

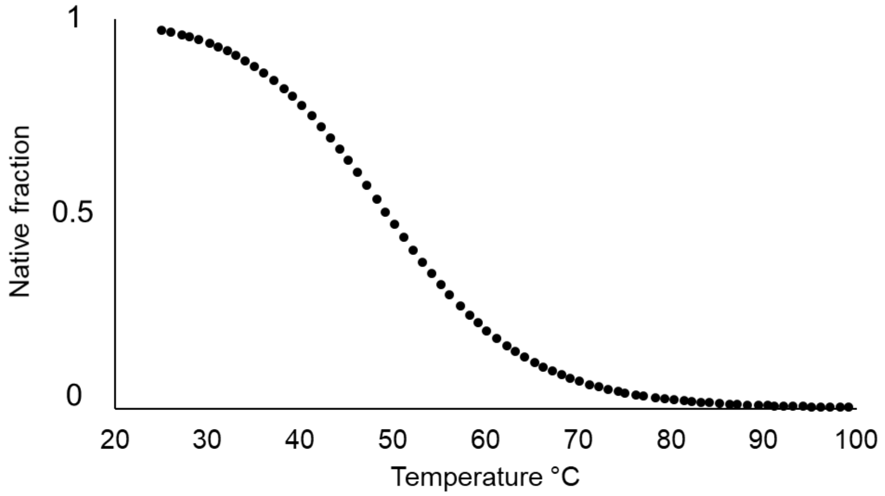

Figure S3. Thermal stability of Ti-CP monitored by circular dichroism (CD). Thermal unfolding profile of Ti-CP obtained by monitoring the ellipticity at 215 nm during a temperature ramp from 25 to 100 °C. The native fraction was calculated from the

normalized ellipticity values as a function of temperature. The sigmoidal transition indicates cooperative thermal denaturation of the protein. The melting temperature (Tm) was determined by fitting the data using the Thermal Denaturation tool available on the BestSel platform.

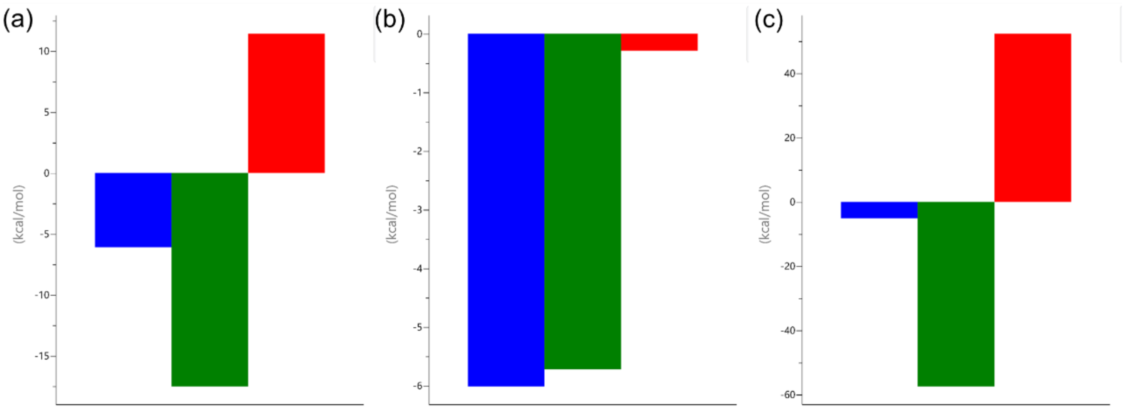

Figure S4. Thermodynamic signature plots for antibiotic binding to Ti-CP determined by isothermal titration calorimetry (ITC). Bar plots represent the relative contributions of Gibbs free energy (ΔG, blue), enthalpy change (ΔH, green), and entropic term (−TΔS, red) to the binding process. (A) Oxacillin, (B) Tetracycline, and (C) Streptomycin. Binding of all ligands is primarily driven by favorable enthalpic contributions, with ligand-dependent entropy penalties reflecting differences in molecular size, polarity, and conformational effects during complex formation.

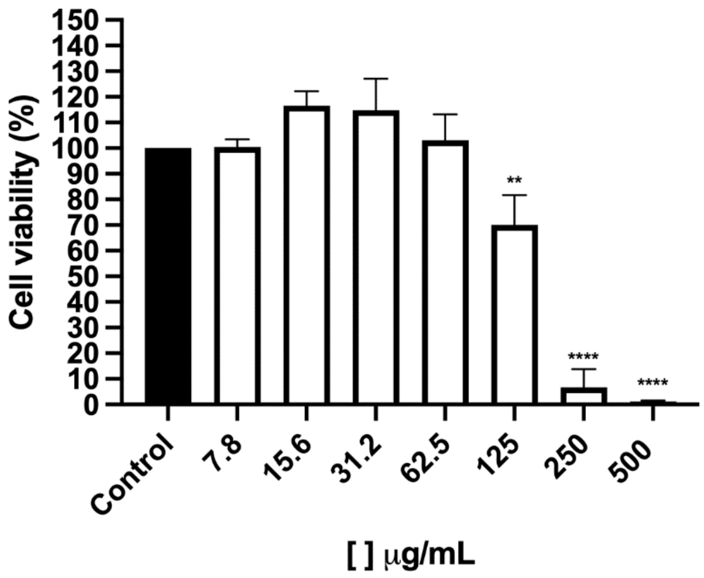

Figure S5. Cytotoxicity assessment of Ti-CP in L929 murine fibroblasts after 48 h of exposure. Cell viability was determined using the MTT assay following treatment with Ti-CP at concentrations ranging from 7.8 to 500 μg·mL^{-1}. Viability is expressed as a

percentage relative to the untreated control. Data are presented as mean ± standard deviation (SD) from three independent biological experiments performed in technical triplicates. Statistical analysis was conducted using one-way ANOVA followed by Tukey's multiple comparisons test. Statistical significance relative to the untreated control is indicated as **$p < 0.01$ and ****$p < 0.0001$.

Table S1. Combined effects of Ti-CP with antibiotics against bacterial strains

**Bacterial strain**	**Antibiotic**	**MIC (alone) (μg/mL)**	**MIC (combined) (μg/mL)**	**MIC ratio**	**Interaction**
Staphylococcus aureus ATCC 25923	Oxacillin	500	250	1/2	Additive
Staphylococcus aureus ATCC 700698 (MRSA)	Oxacillin	62.5	31.25	1/2	Additive
Escherichia coli ATCC 11303	Oxacillin	125	>125	>1	Antagonistic
Staphylococcus aureus ATCC 25923	Tetracycline	0.976	0.244	1/4	Synergistic
Staphylococcus aureus ATCC 700698 (MRSA)	Tetracycline	62.5	15.625	1/4	Synergistic
Escherichia coli ATCC 11303	Tetracycline	7.8	1.95	1/4	Synergistic
Staphylococcus aureus ATCC 25923	Streptomycin	15.625	15.625	1	No interaction
Staphylococcus aureus ATCC 700698 (MRSA)	Streptomycin	7.8	1.95	1/4	Synergistic
Escherichia coli ATCC 11303	Streptomycin	15.625	3.906	1/4	Synergistic

Minimum inhibitory concentration (MIC) values were determined for antibiotics alone (MIC alone) and in combination with Ti-CP (MIC combined). MIC ratios were calculated as MIC combined / MIC alone. Interaction profiles were classified as follows: **1/2,**

additive effect; **≤1/4**, synergistic effect; **=1**, no interaction; **>1**, antagonistic effect. MRSA: methicillin-resistant *Staphylococcus aureus*.

Table S2. Combined effects of ApoTi-CP with antibiotics against bacterial strains

**Bacterial strain**	**Antibiotic**	**MIC alone (µg/mL)**	**MIC combined (µg/mL)**	**MIC ratio**	**Interaction**
Staphylococcus aureus ATCC 25923	Oxacillin	500	250	1/2	Additive
Staphylococcus aureus ATCC 700698 (MRSA)	Oxacillin	62.5	15.625	1/4	Synergistic
Escherichia coli ATCC 11303	Oxacillin	125	>125	>1	Antagonistic
Staphylococcus aureus ATCC 25923	Tetracycline	0.976	0.244	1/4	Synergistic
Staphylococcus aureus ATCC 700698 (MRSA)	Tetracycline	62.5	15.625	1/4	Synergistic
Escherichia coli ATCC 11303	Tetracycline	0.488	0.122	1/4	Synergistic
Staphylococcus aureus ATCC 25923	Streptomycin	15.625	7.8	1/2	Additive
Staphylococcus aureus ATCC 700698 (MRSA)	Streptomycin	7.8	3.9	1/2	Additive
Escherichia coli ATCC 11303	Streptomycin	15.625	7.8	1/2	Additive

Minimum inhibitory concentration (MIC) values were determined for antibiotics alone (MIC alone) and in combination with ApoTi-CP (MIC combined). MIC ratios were calculated as MIC combined / MIC alone. Interaction profiles were classified as follows: **1/2**, additive effect; **≤1/4**, synergistic effect; **=1**, no interaction; **>1**, antagonistic effect. MRSA: methicillin-resistant *Staphylococcus aureus*.
